# Supplementary material for: How human infrastructure threatens biodiversity by squeezing sandy coasts
Source: Curr Biol. 2025 Nov 3;35(21):5210–5219.e2. doi: 10.1016/j.cub.2025.09.027 (PMC12591219; doi:10.1016/j.cub.2025.09.027)
Supplement: Document S1. Figures S1–S3 and Tables S1–S4 [file mmc1.pdf]

Current Biology, Volume *35*

Supplemental Information

How human infrastructure threatens biodiversity by squeezing sandy coasts

Eva M. Lansu, Hallie S. Fischman, Christine Angelini, Nadia Hijner, Luc Geelen, Dick Groenendijk, Solveig Höfer, Annemieke M. Kooijman, Max Rietkerk, Sten Tonkens, Sierd de Vries, Martin Wassen, Evaline van Weerlee, Daniël Wille, Valérie Reijers, and Tjisse van der Heide

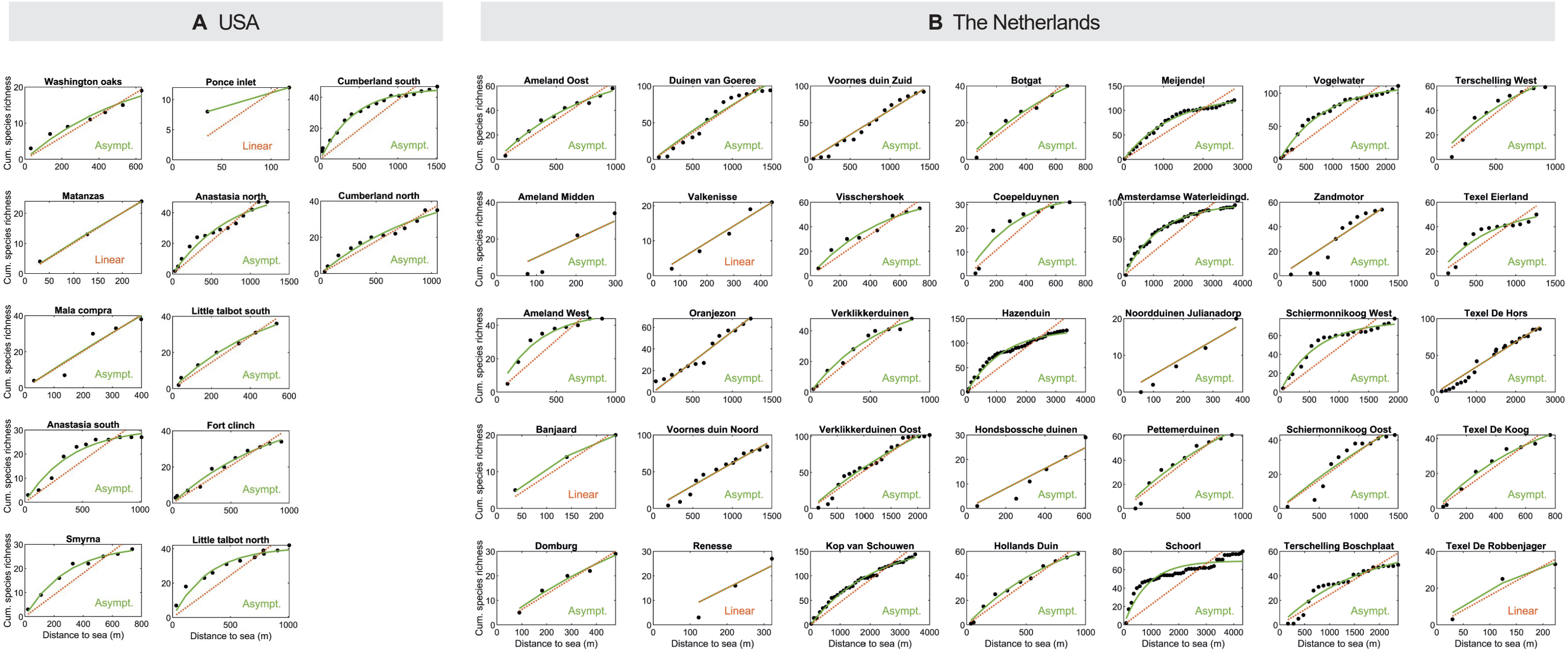

Figure S1. Species accumulation curves per transect. Related to Figure 3. Species accumulation curves of each of the studied transects in southeastern USA (**A**) and the Netherlands (**B**), with the linear regressions (without intercept) displayed orange and the asymptotic regressions displayed green. We selected the best model for each transect based on AIC.

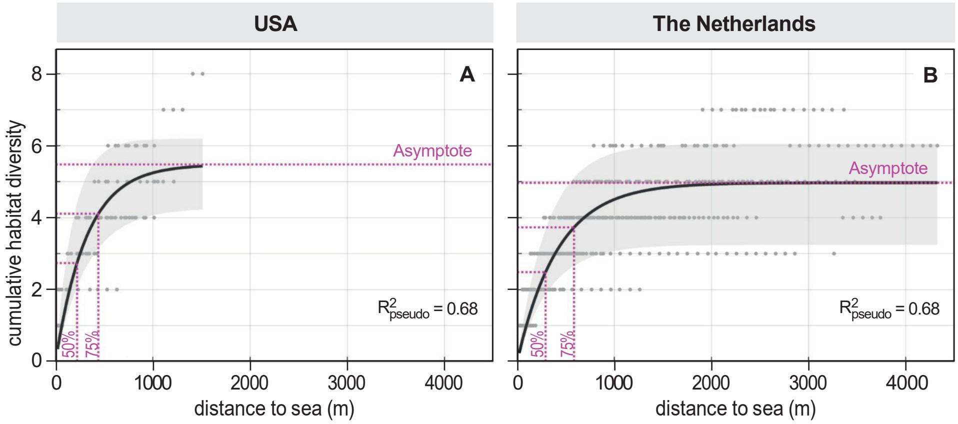

Figure S2. Habitat accumulation curves. Related to Figure 3. Habitat accumulation along the transects in southeastern USA (**A**) and the Netherlands (**B**).

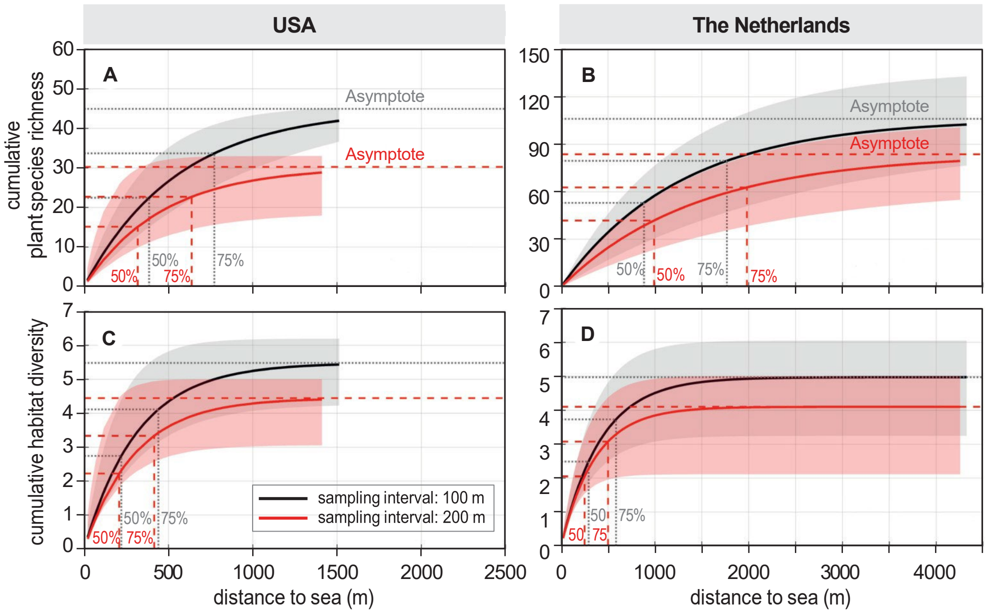

Figure S3. Sensitivity analysis to explore the effect of sampling effort. Related to Figure 3. By deleting every other sample station location along the transects, we increased the sampling interval from 100 m (black line) to 200 m (red line). Subsequently, the species accumulation curves were computed again for the southeastern USA (**A**) and the Netherlands (**B**). In addition, the habitat accumulation curves were computed for both the 100 m and the 200 m interval for the southeastern USA (**C**) and the Netherlands (**D**).

Transects	Transect length (m)	Latitude	Longitude	Species richness	Habitat diversity
Cumberland North	1052	30.78705	–81.45524	49	5
Cumberland South	1512	30.71845	–81.44907	69	8
Fort Clinch	933	30.69228	–81.42811	44	6
Little Talbot North	1008	30.46248	–81.41209	61	5
Little Talbot South	535	30.43905	–81.40802	46	4
Anastasia North	1216	29.90410	–81.27808	67	5
Anastasia South	1013	29.86415	–81.26710	34	4
Matanzas	240	29.70896	–81.22754	38	3
Washington Oaks	631	29.63439	–81.19835	25	2
Mala Compra	399	29.61447	–81.18939	43	3
Ponce Inlet	118	29.08091	–80.92157	19	2
Smyrna	736	29.07317	–80.91648	35	5
Schiermonnikoog Oost	1457	53.509815	6.314283	43	4
Schiermonnikoog West	1940	53.499904	6.167723	78	5
Ameland Oost	974	53.467320	5.910009	58	5
Ameland Midden	298	53.462008	5.801228	34	3
Ameland West	886	53.456472	5.620974	44	3
Terschelling Boschplaat	2374	53.429402	5.411078	49	5
Terschelling West	915	53.401986	5.244782	59	5
Texel De Robbenjager	223	53.182479	4.859673	33	2
Texel Eierland	1262	53.174530	4.832611	50	2
Texel De Koog	766	53.087154	4.740117	42	3
Texel De Hors	2610	52.993224	4.729137	86	7
Noordduinen Julianadorp	378	52.882804	4.708658	20	4
Botgat	677	52.865850	4.702635	40	4
Pettemerduinen	910	52.784281	4.665458	61	5
Hondsbossche duinen	608	52.718910	4.636628	29	3
Schoorl	4325	52.700660	4.635027	80	6
Vogelwater	2231	52.586610	4.612882	111	6
Hazenduin	3364	52.534578	4.599579	126	7
Amsterdamse Waterleidingduinen	3741	52.315653	4.484371	96	4
Hollands Duin	937	52.272737	4.451405	58	5
Coepelduynen	693	52.225119	4.410146	31	4
Meijendel	2790	52.155730	4.338499	121	5
Zandmotor	1294	52.049719	4.181619	54	4
Voornes duin Noord	1440	51.910601	4.048451	84	5
Voornes duin Zuid	1425	51.891549	4.030823	92	5
Duinen van Goeree	1479	51.836959	3.942686	94	6
Visschershoek	733	51.788766	3.863601	55	4
Renesse	321	51.743192	3.766741	27	2
Verklikkerduinen Oost	2224	51.733573	3.712875	102	4
Verklikkerduinen	849	51.720931	3.690537	48	4
Kop van Schouwen	3512	51.691457	3.681640	144	6
Banjaard	239	51.594930	3.665045	20	2
Oranjezon	1232	51.592296	3.567357	68	5
Domburg	477	51.574618	3.517485	29	3
Valkenisse	441	51.485731	3.504876	21	3

Table S1. Details of transects. Related to Figure 2. Details of transects in southeastern USA and the Netherlands.

**Southeastern USA**	
Broadleaf forest	– Tree cover > 25% AND:<br>○ EITHER: The dominant tree species is *Quercus virginiana*<br>○ OR: Needleleaf tree cover < 50% AND Palm tree cover < 50%
Salt Marsh	– Salt loving species cover > 25% OR (salt loving species cover / total herb cover) > 50%
Needleleaf forest	– Tree cover > 25%<br>– AND Needleleaf tree cover > 50%
Open dune grassland	– Tree cover < 25% AND Shrub cover < 25% AND (Shrub + Tree cover) < 25%<br>– AND total vegetation cover < 50%
Palm forest	– Tree cover > 25%<br>– AND Palm tree cover > 50%
Shrubs	– Shrub cover > 25% OR (Shrub + Tree cover) > 25%
Closed dune grassland	– Tree cover < 25% AND Shrub cover < 25% AND (Shrub + Tree cover) < 25%<br>– AND: total vegetation cover > 50%
Foredune	– The seaward–most established (non–incipient) dune; based on field observation
Embryo dune	– Incipient dune on the beach; based on field observation

**The Netherlands**	
Heather	– Tree cover < 25*% AND:*<br>○ EITHER: *Empetrum nigrum* is present<br>○ OR: *Calluna vulgaris* > 25% OR (*Calluna vulgaris* / total herb cover) > 50%
Salt Marsh	– Based on field observation of salt marsh
Needleleaf forest	– Tree cover > 25%<br>– AND: Needleleaf tree cover > 50%
Broadleaf forest	– Tree cover > 25%<br>– AND: Needleleaf tree cover < 50%
Dune Slack	– Dune slack species cover > 25% OR (dune slack species / total herb cover) > 50%<br>– OR: Based on field observation
Closed dune grassland	– Tree cover < 25% AND Shrub cover < 25% AND (Shrub + Tree cover) < 25%<br>– AND: total vegetation cover > 50%
Shrubs	– Shrub cover > 25% OR (Shrub + Tree cover) > 25%
Open dune grassland	– Tree cover < 25% AND Shrub cover < 25% AND (Shrub + Tree cover) < 25%<br>– AND: total vegetation cover < 50%
Foredune	– The seaward–most established (non–incipient) dune; based on field observation
Embryo dune	– Incipient dune on the beach; based on field observation

Table S2. Habitat criteria. Related to STAR Methods. The criteria used to assign habitat types to the sample stations in southeastern USA and the Netherlands.

Transects in southeastern USA	Number of managements interventions	Controlled burns	Invasive plant species removal	Invasive animal species removal	Grazing
Cumberland North	2	0	0	1	1
Cumberland South	3	0	1	1	1
Fort Clinch	2	0	1	1	0
Little Talbot North	2	0	1	1	0
Little Talbot South	2	0	1	1	0
Anastasia North	3	1	1	1	0
Anastasia South	3	1	1	1	0
Matanzas	2	0	1	1	0
Washington Oaks	1	0	1	0	0
Mala Compra	1	0	1	0	0
Ponce Inlet	1	0	1	0	0
Smyrna	1	0	1	0	0

Transects in The Netherlands	Number of management interventions	Sod cutting	Mowing	Flailing	Grazing	Shrub removal	Creation of notches	Creation of blow- outs
Schiermonnikoog Oost	0	0	0	0	0	0	0	0
Schiermonnikoog West	3	0	1	1	0	1	0	0
Ameland Oost	3	1	0	0	0	1	1	0
Ameland Midden	1	0	0	0	0	0	1	0
Ameland West	0	0	0	0	0	0	0	0
Terschelling Boschplaat	0	0	0	0	0	0	0	0
Terschelling West	3	1	1	0	1	0	0	0
Texel De Robbenjager	1	0	1	0	0	0	0	0
Texel Eierland	4	0	1	1	1	0	0	1
Texel De Koog	2	0	0	0	1	1	0	0
Texel De Hors	1	0	1	0	0	0	0	0
Noordduinen Julianadorp	3	0	1	0	0	1	0	1
Botgat	0	0	0	0	0	0	0	0
Pettemerduinen	4	0	1	1	1	1	0	0
Hondsbossche duinen	0	0	0	0	0	0	0	0
Schoorl	5	1	0	1	1	1	0	1
Vogelwater	5	0	1	0	1	1	1	1
Hazenduin	4	0	1	0	1	1	0	1
Amsterdamse Waterleidingd.	6	1	1	0	1	1	1	1
Hollands Duin	2	0	0	0	0	1	0	1
Coepelduynen	0	0	0	0	0	0	0	0
Meijendel	5	1	1	0	1	1	1	0
Zandmotor	3	0	0	0	1	1	1	0
Voornes duin Noord	1	0	0	0	0	1	0	0
Voornes duin Zuid	3	0	1	0	1	1	0	0
Duinen van Goeree	4	0	1	1	1	1	0	0
Visschershoek	5	0	1	0	1	1	1	1
Renesse	2	0	0	0	1	1	0	0
Verklikkerduinen Oost	3	0	1	0	1	1	0	0
Verklikkerduinen	3	1	0	0	1	1	0	0
Kop van Schouwen	4	1	0	0	1	1	1	0
Banjaard	0	0	0	0	0	0	0	0
Oranjezon	3	1	0	0	1	1	0	0
Domburg	3	0	1	1	0	1	0	0
Valkenisse	1	0	1	0	0	0	0	0

Table S3. Result of management questionnaires. Related to Table 1. Result of questionnaires about management interventions targeted at resetting vegetation succession and increasing sediment dynamics in southeastern USA and in the Netherlands.

The Netherlands

Linear regression model: species richness ~ 1 + transect length * number of interventions per km transect

	St. Estimate	SE	P value
**Intercept**	0.11	0.15	0.44
**Transect length**	0.62	0.15	2.1E-04
**Number of interventions per km transect**	0.60	0.19	3.8E-03
**Transect length: Number of interventions per km transect**	0.48	0.21	3.1E-02

Number of observations: 35

R-squared: 0.40

Adjusted R-Squared: 0.34

Table S4. Regression model. Related to Table 1. Linear regression model to explain species richness of the Dutch transects. The species richness was subsampled to ensure uniform sampling effort across transects. To approach a normal distribution of regression residuals, the explaining variables were square root transformed. Additionally, all variables were standardized using z-score transformation.
