# Supplementary material for: How human infrastructure threatens biodiversity by squeezing sandy coasts
Source: Curr Biol. 2025 Nov 3;35(21):5210–5219.e2. doi: 10.1016/j.cub.2025.09.027 (PMC12591219; doi:10.1016/j.cub.2025.09.027)
Supplement: Document S2. Article plus supplemental information [file mmc2.pdf]

Current Biology

Article

How human infrastructure threatens biodiversity by squeezing sandy coasts

Graphical abstract

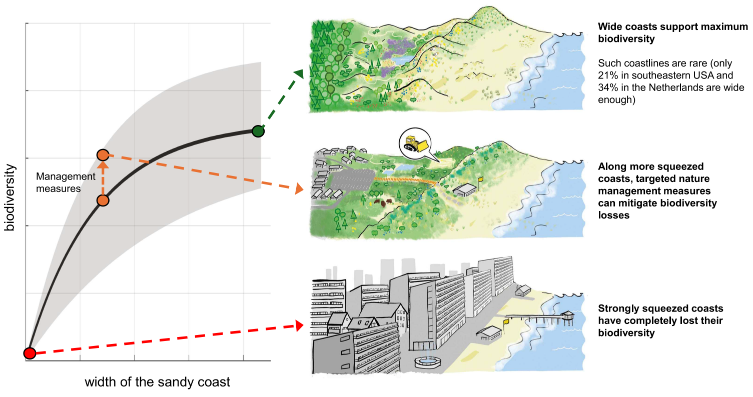

Authors

Eva M. Lansu, Hallie S. Fischman, Christine Angelini, ..., Daniël Wille, Valérie Reijers, Tjisse van der Heide

Correspondence

eva.lansu@nioz.nl

In brief

Lansu et al. show that dune biodiversity in the Netherlands and southeastern USA increases with undisturbed coastal width, reaching 75% of its potential at 800–1,800 m. Alarmingly, most coasts are much narrower. To conserve biodiversity, it is essential to protect remaining dune systems, while management can partly mitigate losses.

Highlights

- Plant biodiversity increases nonlinearly with increasing undisturbed coastal width
- Biodiversity reaches 75% of its max at 800 (US) to 1,800 m (the Netherlands) coastal width
- Infrastructure squeezes 79% of US and 66% of Dutch coasts into much narrower strips
- Management can partly mitigate infrastructure-driven biodiversity losses

Lansu et al., 2025, Current Biology *35*, 5210–5219
November 3, 2025 © 2025 The Authors. Published by Elsevier Inc.
https://doi.org/10.1016/j.cub.2025.09.027

Check for updates
CellPress

CellPress
OPEN ACCESS

Current Biology

Article

How human infrastructure threatens biodiversity by squeezing sandy coasts

Eva M. Lansu,[1,2,10,*] Hallie S. Fischman,[3] Christine Angelini,[3] Nadia Hijner,[2] Luc Geelen,[4] Dick Groenendijk,[5] Solveig Höfer,[1,2] Annemieke M. Kooijman,[6] Max Rietkerk,[7] Sten Tonkens,[1] Sierd de Vries,[8] Martin Wassen,[7] Evaline van Weerlee,[1] Daniël Wille,[1] Valérie Reijers,[9] and Tjisse van der Heide[1,2]

[1]Department of Coastal Systems, Royal Netherlands Institute for Sea Research (NIOZ), Den Burg, Texel 1790 AB, the Netherlands
[2]Conservation Ecology Group, Groningen Institute for Evolutionary Life Sciences, University of Groningen, Groningen 9700 CC, the Netherlands
[3]Center for Coastal Solutions, Engineering School for Sustainable Infrastructure and Environment, University of Florida, Gainesville, FL 32611, USA
[4]Waternet, Vogelenzang 2114 BH, the Netherlands
[5]PWN Waterleidingbedrijf Noord-Holland, Velserbroek 1991 AS, the Netherlands
[6]Department of Ecosystem and Landscape Dynamics, Institute of Biodiversity and Ecosystem Dynamics, University of Amsterdam, Amsterdam 1098 XH, the Netherlands
[7]Copernicus Institute of Sustainable Development, Section Environmental Science, Utrecht University, Utrecht 3584 CB, the Netherlands
[8]Department of Hydraulic Engineering, Faculty of Civil Engineering and Geosciences, Delft University of Technology, Delft 2600 GA, the Netherlands
[9]Department of Physical Geography, Faculty of Geosciences, Utrecht University, Utrecht 3584 CB, the Netherlands
[10]Lead contact
*Correspondence: eva.lansu@nioz.nl
https://doi.org/10.1016/j.cub.2025.09.027

SUMMARY

Coastal dunes form valuable ecosystems that provide flood protection, drinking water, and high biodiversity worldwide. Although their functioning hinges on habitat zonation along >km-scale sea-to-land gradients, infrastructure development progressively squeezes natural dune ecosystems into a narrow strip. Yet it remains unknown how much undisturbed coastal width is required to support the diverse suites of habitats and species assemblages found in natural dune systems. Here, we investigate plant and habitat diversity in 614 plots along 47 sea-to-land transects in the southeastern USA and the Netherlands. We discover a linear relation between habitat diversity and species richness, indicating that species-rich dunes require diverse habitat assemblages. Moreover, we find that both plant and habitat diversity nonlinearly depend on coastal width, with cumulative plant diversity reaching ∼75% of its potential at 800 and 1,800 m widths in the southeastern USA and the Netherlands, respectively. Alarmingly, dune areas are narrower than these widths along 79% and 66% of southeastern USA and Dutch coastlines, highlighting that lack of space compromises biodiversity along the majority of coastlines. Finally, analyses of management measures along the transects reveal that strategic interventions can, at least in part, mitigate biodiversity losses from infrastructure encroachment. As coastal squeeze—i.e., combined losses from infrastructure and sea level rise—is a global phenomenon, our results suggest that it threatens biodiversity in dune ecosystems worldwide. We argue that the establishment or expansion of nature reserves may be vital for conserving wide dune systems and that targeted management measures can help maintain biodiversity where squeeze cannot be alleviated.

INTRODUCTION

About one-third of the world's shoreline is sandy, and many of these sandy coasts support developed dune ecosystems.[1,2] These beach-dune landscapes provide many ecosystem services to humanity, which include flood protection, carbon storage, recreation, freshwater storage, and biodiversity.[3–6] As the global coastal population continues to expand,[7,8] humans increasingly depend on these services. However, anthropogenic pressures—including infrastructure development, eutrophication, and pollution—are increasingly degrading beach-dune ecosystems worldwide.[6,9–13]

To generate ecosystem services, dunes require space to establish and biogenically build complex topography. A prime example is the requirement for a sufficiently wide beach to support the development of embryo dunes, as highlighted by the 300-meter benchmark identified for European beaches.[14–16] On these wide beaches, embryo dunes form the onset of more developed dune formations, which further widen the dune landscape.[15,17] In a wide dune system, sand erosion and deposition generally decrease in a landward direction, supporting the establishment of less disturbance-tolerant species.[18–20] Superimposed upon these large-scale morphological gradients, dune formation gives rise to variations in solar radiation, soil

 © 2025 The Authors. Published by Elsevier Inc.
This is an open access article under the CC BY license (http://creativecommons.org/licenses/by/4.0/).

water content, and organic matter across dune slopes and valleys.[17,18,21] Overall, the combination of large- and small-scale gradients results in a habitat-rich dune landscape. In the relatively humid temperate oceanic climate, this landscape typically consists of a sea-to-land sequence of embryo dunes, foredunes, dune grasslands, dune slacks, shrublands, and forests (Figure 1A). To support this habitat-rich landscape, intact sea-to-land gradients are essential, and these gradients in turn require sufficient space to develop.

Human activities often confine the space required for the self-organized formation of coastal dunes. In particular, hard structures (e.g., roads and buildings in the coastal zone) truncate the continuity of large-scale gradients and thus the eco-geomorphology of the dune landscape. Such manmade structures lead to direct habitat loss and habitat fragmentation and hinder the ability of the landscape to adapt to sea level rise. The combination of encroaching infrastructure and a rising sea is a phenomenon known as "coastal squeeze."[6,22–24] Moreover, infrastructure development may hamper dispersal and recolonization and induce adverse edge effects, thereby driving biodiversity losses.[25–27] As infrastructure development was recently found to truncate sandy coasts worldwide to a median width of just 392 m globally, this narrowing of sandy coasts stands out as one of the most pervasive threats to the functioning coastal dune landscapes (Figure 1).[28] Yet thus far it remains unknown how squeeze by human infrastructure affects habitat and species diversity in coastal dune landscapes.

Here, we study how the undisturbed coastal width affects dune habitat diversity and plant species richness and quantify the minimal unimpacted space required to support a biodiverse dune ecosystem. We define the undisturbed coastal width as the distance between the highwater line and the nearest human infrastructure (paved road and/or building). We thus consider dune areas with a narrower undisturbed coastal width (i.e., infrastructure closer to shore) more "squeezed," as they are confined between human infrastructure on one side and a rising sea on the other. As many coasts worldwide are already heavily squeezed by human infrastructure, we next evaluate the potential of management strategies to enhance species richness through surveys of coastal managers. We hypothesize that (1) coastal width is an important determinant for both habitat diversity and species richness and (2) species richness saturates at a certain coastal width. The latter hypothesis is in line with Barbier et al.[29] and Koch et al.,[30] who found that the ecological functioning of coastal habitats increases nonlinearly with ecosystem size. Furthermore, we expect targeted management measures to be able to increase species richness in narrower dune systems, thereby (partly) mitigating adverse effects of coastal squeeze.

To test our hypotheses, we examined the relationship between coastal width and plant species richness in two distinct coastal dune landscapes on both sides of the Atlantic Ocean: the temperate oceanic coast of the Netherlands and the humid subtropical coastline of Florida and Georgia (hereafter: southeastern USA). We performed 506 vegetation recordings at 100-m intervals along 35 cross-shore transects in the Netherlands and 108 recordings within 12 transects in the southeastern USA (Figure 2). Next, we analyzed the relation between coastal width and plant species richness and habitat diversity, respectively, by fitting species accumulation curves for both the southeastern USA and the Netherlands. Indeed, previous work demonstrated that the local species richness in a specific habitat depends on the local (patch) size of that specific habitat type. This dependency has been shown in several habitats, e.g., dune slacks, embryo dunes, and mobile dunes.[31–33] Here, we use species-area relationships in a novel way to explore how coastal squeeze reduces plant biodiversity at the landscape scale by shrinking and eliminating entire habitats in both temperate and subtropical dune systems. To explore whether management measures can increase species richness and mitigate narrowing of the natural coast, we conducted surveys among nature managers to determine the number of measures taken along each transect. Finally, we fitted the total species richness per transect to the number of measures per km transect and the overall transect length using stepwise linear regression.

RESULTS

Species richness versus coastal width

Regression analyses of the cumulative plant species richness across the sea-to-land transects reveal that the number of plant species accumulates nonlinearly with increasing distance to sea in both the southeastern USA and the Netherlands (Figure 3). Examining individual transects shows that 10 out of 12 transects in the USA and 31 out of 35 transects in the Netherlands follow this asymptotic, saturating pattern, with only the shortest transects (200–400 m) being better described by a linear regression model (Figure S1). In the southeastern USA, transects were generally shorter than in the Netherlands, as buildings and paved roads are closer to the shoreline in this region.[28] Whereas the combined USA regression curve (Figure 3A) describes the species accumulation up to 1,500 m, the regression curve from the Netherlands (Figure 3B) describes the species accumulation up to 4,300 m from the sea. Nevertheless, in both study areas, cumulative species richness across the combined transects is better described by an asymptotic regression than by a linear regression (ΔAIC: 15 and 213 in USA and NL, respectively).

The asymptotic regressions (Figures 3A and 3B) on the combined transect data explain 82% of the observed variance in species richness in the southeastern USA and 74% in the Netherlands. The asymptotic value of the regression curve (i.e., the potential maximum number of species) is estimated at 45 plant species (95% confidence interval [CI]: 39–51 species) in the USA. This value is approached (i.e., 99% is reached) at approximately 2,600 m from the sea, while 50% and 75% of this maximum are reached after 400 and 800 m, respectively (Figure 3A). In the Netherlands, the combined regression curve maxes out at 106 plant species (95% CI: 100–112 species), which is approached (i.e., 99% is reached) at approximately 5,900 m from the sea, with 50% reached after 900 m and 75% after 1,800 m (Figure 3B). Finally, the initial relative increase of species richness (proportional to the maximum) is greater in the USA than in the Netherlands (USA, 1.8e−03, 95% CI 1.3e−03–2.2e−03; NL, 7.8e−04, 95% CI 6.9e−04–8.8e−04). In summary, the species accumulation curve from the USA approaches its maximum at a two-times shorter distance to sea, and this maximum equals about half the Dutch maximum.

To explore the extent to which sampling effort affected our results, we performed sensitivity analyses in which we increased

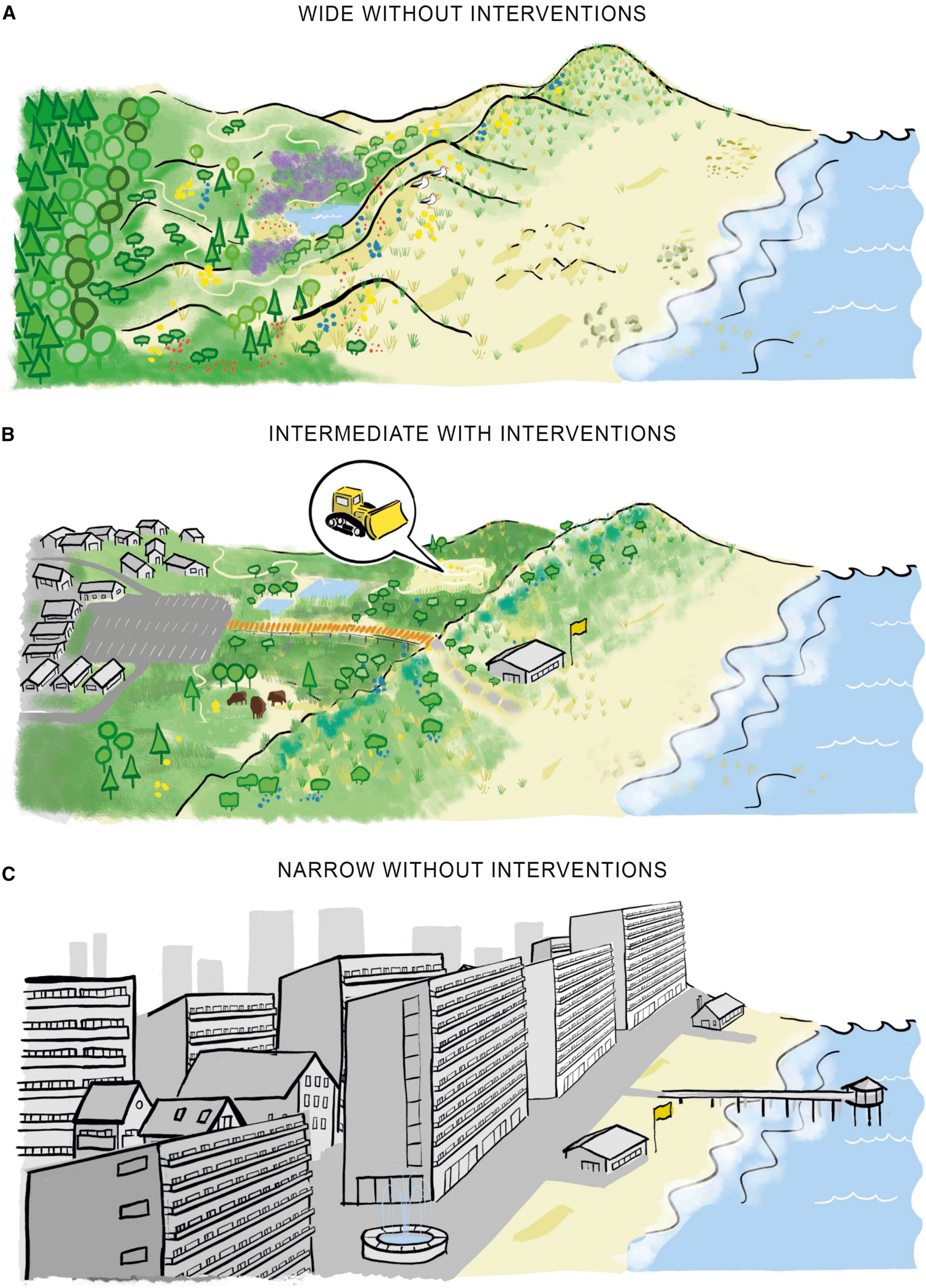

Figure 1. Conceptual overview

An intact dune ecosystem with a self-organized, habitat-rich landscape (A), a partly degraded dune system—encroached by human infrastructure—where management interventions increase habitat diversity and species richness (B), and a heavily degraded system where management interventions are not effective anymore (C).

CellPress
OPEN ACCESS

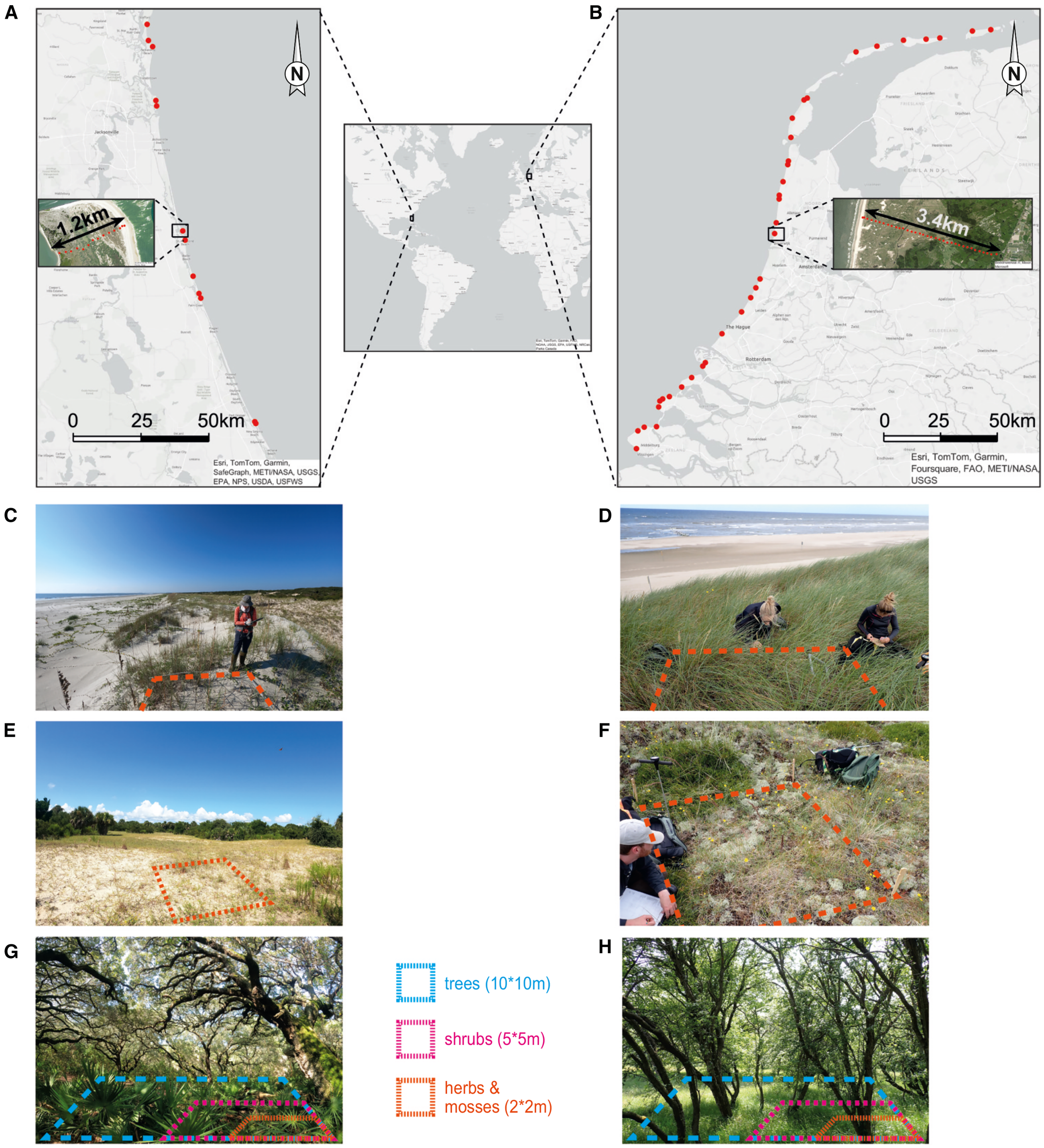

Figure 2. The coastal dune sites

(A and B) We sampled along sea-to-land transects in the southeast of the USA (A) and the Netherlands (B). The inserted maps show the 100 m-spaced sampling stations of a transect (coordinates in Table S1).

(C–H) Examples of such sampling stations are in the southeast of the USA (C, E, and G) and the Netherlands (D, F, and H). At each sampling station, we identified herb and moss species in a 2 × 2 m plot. If present, shrub and tree species were identified in 5 × 5 and 10 × 10 m plots, respectively.

Table S1

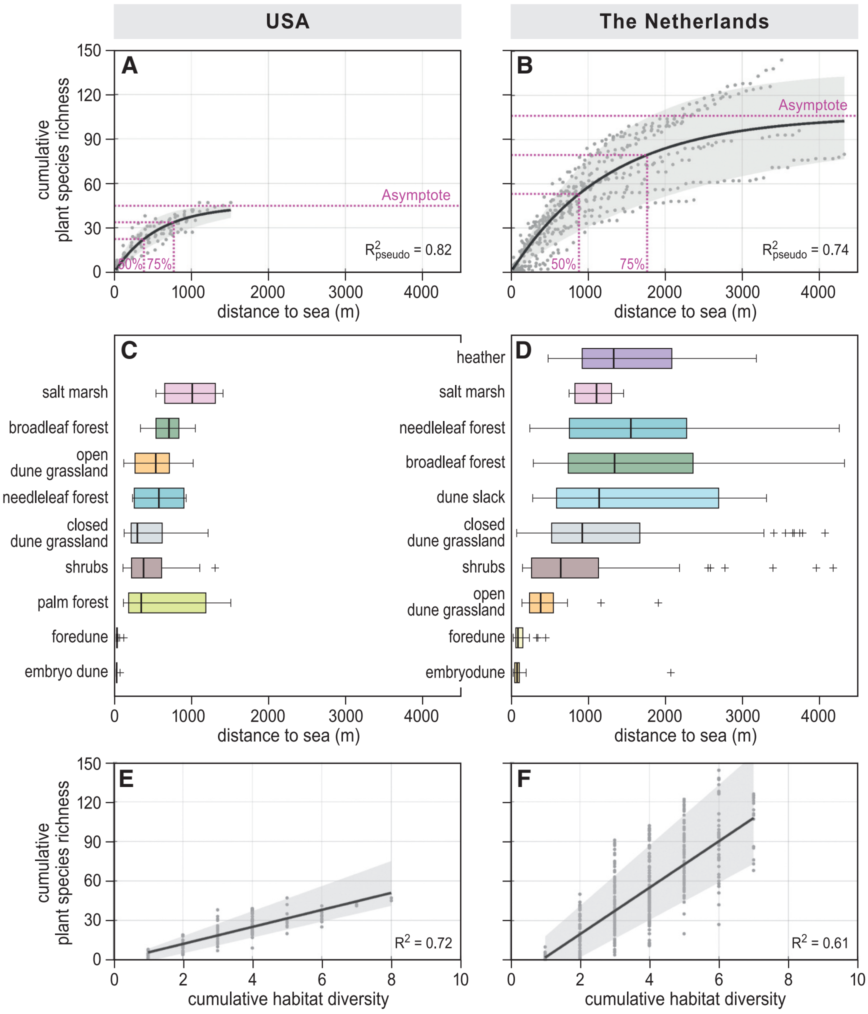

Figure 3. Field observations of species richness and habitat diversity

Plant species accumulation as a function of distance to sea with the fitted asymptotic regression (black line) and its CI between the 10th and 90th percentile (gray area) (A and B), the distance to sea at which habitats occur (C and D), and cumulative plant species along the transects as a function of the accumulated number of habitats with the fitted asymptotic regression (black line) and its CI between the 10th and 90th percentile (gray area) (E and F) for the southeastern USA and the Netherlands, respectively.

See also Figures S1–S3.

the sampling interval from 100 to 200 m by removing observations from the dataset (Figure S3). As expected, these analyses show that doubling the sampling interval reduces the asymptote of the species richness curves by 33% in the southeastern USA and by 21% in the Netherlands. However, the required width to reach 75% of the species changed much less, with a 17% decrease in the USA and a 12% increase in the Netherlands (Figures S3A and S3B). Similarly, the required width to reach 50% of the species maximum shifts with the same proportion (i.e., USA −17%; NL +12%). Apart from the species accumulation curve, the habitat accumulation curve also changed marginally when doubling the sampling interval (Figures S3C and S3D). Specifically, the required width to reach both 75% and 50% of the habitat maximum in both study areas decreases by 6% in the USA and 15% in the Netherlands.

To investigate the potential driving role of habitat diversity, we computed the distance between the sea and the first appearance of each habitat (with respect to the sea). In line with the steeper accumulation of species, habitats along the southeastern USA coastline generally first appear closer to the sea (Figure 3C), with the accumulation curves following a nonlinear response with increasing distance from the sea (Figure S2A). The Dutch dune habitats have a larger variance in their first occurrence but also follow a nonlinear increase with distance (Figures 3D and S2B), highlighting their greater spread over the sea-to-land gradient. Further analyses reveal that habitat and species richness follow a strong linear correlation in both studied areas (USA R^2 0.72, NL R^2 0.61; Figures 3E and 3F). These findings suggest that species richness heavily depends on the number of habitats present along the transect.

Mapping species richness potential

We applied the above-described species richness versus coastal width models (Figures 3A and 3B) to assess the species richness along the Dutch and southeastern USA coastlines. Specifically, we used the infrastructure-free coastal width as a measure for unimpacted space[28] and calculated the percentage of the species richness that can potentially be reached within the available unimpacted coastal width, using the developed nonlinear functions. Results highlight that 21% of the USA sandy coastline is sufficiently wide (800 m) to accommodate more than 75% of the species richness potential, while 26% is sufficiently wide (400 m) to reach at least 50% of the potential. In the Netherlands, 34% of the sandy coast is wide enough (1,800 m) to accommodate over 75% of the species richness potential, and 55% is sufficiently wide (900 m) to host at least 50% of this potential (Figure 4). Whereas a large part of the Dutch sandy coastline (87%) is protected by nature reserves, only 35% of the southeastern USA's sandy shoreline has a protected status. Moreover, we find that typically the wider unimpacted sandy coasts are located in protected areas.[28] Specifically, 77% and 92% of the wide (wide enough to harbor 75% of the maximum species richness potential) sandy coasts in the southeastern USA and the Netherlands, respectively, are located in protected areas.

Management interventions

We carried out surveys among nature managers of the areas that encompassed all 35 Dutch and 12 southeastern USA transects of our study (Tables 1 and S3). In the Netherlands, seven types of interventions were applied over the course of the last 20 years,

CellPress
OPEN ACCESS

Table 1. Management interventions

Interventions in the USA	Occurrence	Interventions in the Netherlands	Occurrence
Controlled burning	2	sod cutting	8
Invasive plant species removal	11	mowing	17
Invasive animal species removal	8	flailing	6
Grazing	2	grazing	18
–	–	shrub removal	22
–	–	creation of notches	8
–	–	creation of blowouts	8

Measures were taken at the transect sites in the southeastern USA (n = 12) and in the Netherlands (n = 35). We included interventions targeted at resetting vegetation succession and increasing sediment dynamics. The numbers indicate the number of transects at which interventions were performed. See also Tables S3 and S4.

while in the USA, four intervention types have been applied over this period. Next, we created a simple stepwise linear regression model, which removed terms based on the AIC, to explain average species richness per transect with transect length and number of management interventions per km of transect as covariates. Results show that in the Netherlands, 40% of the variance in species richness can be explained by the combined effects of transect length and the number of management interventions per km of transect. The standardized model parameters highlight that transect length (0.62) is slightly more important in explaining the species richness variance than the number of management interventions per transect kilometer (0.60) (Table S4). Moreover, the interaction effect of transect length and number of interventions also explains a significant part of the variation in species richness (0.48). In the stepwise linear regression model of the USA, both explanatory variables—transect length and management interventions per km transect—were removed.

DISCUSSION

Our results show that coastal width is a vital determinant of plant species richness along the sandy coasts of both the southeastern USA and the Netherlands. Specifically, cumulative species richness increases in a nonlinear, saturating manner as the dune ecosystem broadens and correlates linearly with the number of habitats along the sea-to-land gradient. Moreover, our work reveals that the calculated 800 and 1,800 m of infrastructure-free space required to accommodate 75% of the species richness in the southeastern USA and the Netherlands, respectively, are not met in most areas along each coastline. While lack of space is eroding biodiversity of dune systems, results from the manager survey show that targeted management interventions can work synergistically with dune conservation via protected areas to mitigate the negative impacts of coastal squeeze in the Netherlands. However, along the even more intensively developed shoreline of the southeastern USA, our results indicate that protection of sufficiently wide dune areas is the most impactful tool to maintain biodiverse dune systems.

How coastal width controls species richness

Following our hypotheses, the plant species richness nonlinearly increased with increasing coastal width, saturating after about 1–2 km. This steep initial accumulation of species richness typically occurs as new habitats emerge, which mainly occurs along the first 500 m along the transects (Figure S2). In line with previous work, we find that species richness linearly correlates with the number of habitats.[34,35] Our results therefore suggest that habitat diversity is the primary driver of plant diversity. In turn, habitat diversity depends on the presence of intact sea-to-land gradients, which require a sufficiently wide coastal zone.[19,21] This underlines the driving role of coastal width for both habitat and plant species diversity. Moreover, as species richness at higher trophic levels is typically closely linked to plant species richness,[36,37] the spatial dependence of species and habitat diversity is likely mirrored by higher trophic levels. Therefore, our findings likely reflect not only the importance of coastal width for the plant community but also for overall biodiversity and ecosystem functioning of coastal dunes.[38–40]

By computing species accumulation curves, species richness can be assessed and compared between areas.[41,42] A drawback of such an approach, however, is that the number of species found in an area depends on the size of each sample and/or the sampling effort,[43–45] which is also the case for our study. Indeed, our sensitivity analyses (Figure S3) show that doubling the sampling interval decreases the richness maximum by 33% in the USA and 21% in the Netherlands, highlighting that reducing the sampling effort lowers the estimated maximum of species richness. Apart from the direct effect of finding a lower number of species due to a lower number of samples, the decreased maximum is also related to the skipping of habitats. Indeed, doubling the sampling interval decreased the average number of habitats found within the transects from 4.3 to 3.4 in the USA and from 4.3 to 3.7 in the Netherlands. Moreover, the points at which 75% of species richness was reached changed only by 12%–17% for both regions, suggesting that these estimates are rather robust. So although absolute values in our outcomes are sensitive to the sampling interval as expected, this does not affect our overall conclusion that coastal width controls habitat and plant diversity in a nonlinear fashion.

This work offers the first quantitative estimates of the minimum undisturbed coastal width required to sustain (a certain level of) biodiversity. By relating coastal width to basic yet robust biodiversity metrics—plant species richness and habitat diversity—we provide a framework to assess how coastal squeeze threatens biodiversity along sandy coasts. Although we found strong relationships between coastal width and our biodiversity metrics, not all variability can be explained by these metrics. This is likely due to additional factors that vary between transects, such as differences in regional species pools and local topography.[46] Future work could expand on our analyses by including such factors and by including longshore spatial patterns rather than merely focusing on cross-shore patterns. In addition, there are also other biodiversity metrics that could be considered, including metrics that indicate potential diversity gaps (e.g., local:dark diversity ratio[27,47]) or that take into account

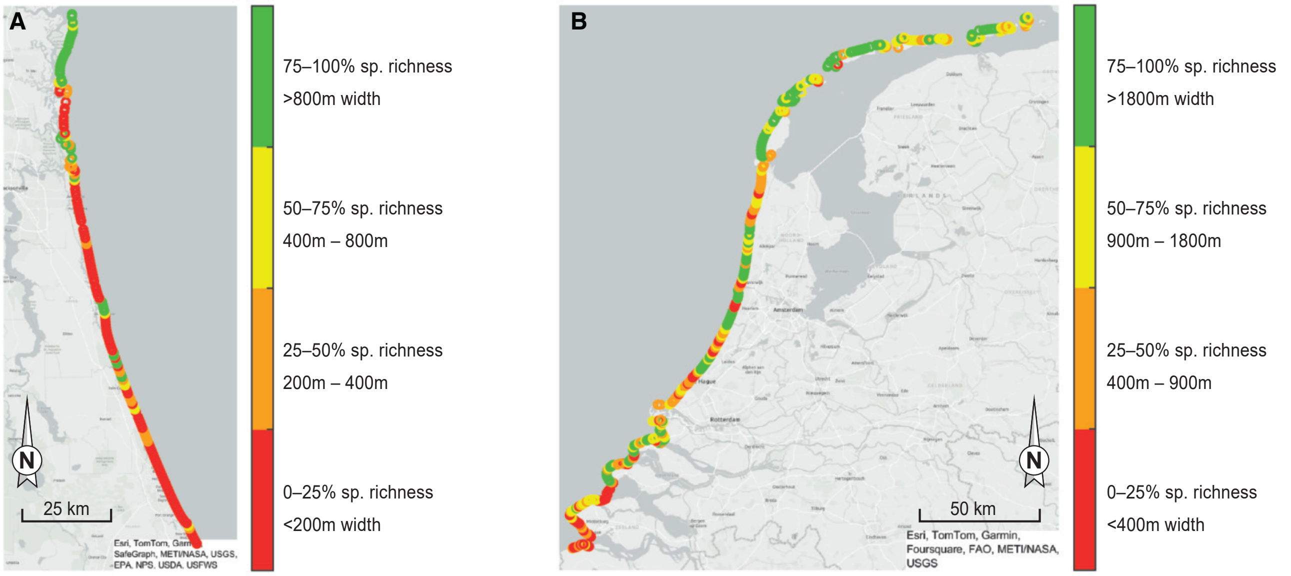

Figure 4. Predicted species richness

The maps show the computed percentage of maximum species richness reached, given the local infrastructure-free width of the sandy coast, for the southeastern USA (A) and the Netherlands (B). In total, 225 km of sandy coastline was assessed in the southeast of the USA, and 425 km in the Netherlands.

species evenness (e.g., Shannon and Simpson index[48]). Yet despite these limitations, our analyses clearly show that coastal squeeze predictably reduces plant diversity by shrinking and eliminating entire habitats across the landscape scale.

Shortage of coastal width

Recent work found that infrastructure development close to shore is a widespread global phenomenon.[28] Human-made structures were found to occur at a median distance of less than 400 m along the world's sandy shores, with one-third having less than 100 m of infrastructure-free space. Here, we find in an empirical transatlantic study that the required minimum width to sustain 75% of the plant diversity in a coastal dune area is at least 800 m in the USA and 1,800 m in the Netherlands. These results suggest that nearshore human infrastructure may severely impact biodiversity in coastal areas worldwide. Indeed, we find that in our study areas, many dune areas are constricted by human infrastructure and too narrow to develop their species richness potential. Specifically, only 21% of the sandy coast along the southeastern USA currently has a wide enough infrastructure-free zone (over 800 m) to accommodate at least 75% of the species richness potential. In the Netherlands, 34% of the unimpacted sandy coast is sufficiently wide (over 1,800 m) to sustain over 75% of the species richness potential (Figure 4). In both study areas, these wide systems are generally located within protected areas. This indicates that while nature reserves alone cannot increase coastal space, they can be important in preventing further infrastructure encroachment.[28] Therefore, since maintaining wide dune systems is the most straightforward way to protect biodiversity, we argue that establishment or expansion of nature reserves encompassing the entire required coastal width may be vital to conserve dunes and their ecosystem services.

How to counter biodiversity losses

Whereas our results highlight that the widest coastal dune systems typically harbor the highest biodiversity, this does not mean that narrower systems truncated by infrastructure do not hold ecological value. Indeed, we found that cumulative species richness steeply increases within the first 1–2 km. This, in turn, means that high conservation gains can be achieved by protecting the areas closest to the sea from infrastructure encroachment. Notably, these findings closely align with earlier work from Barbier et al.,[29] who demonstrated that ecosystem functions, such as wave attenuation, also nonlinearly depend on coastal width. At present, however, coastal populations continue to grow worldwide,[7] with infrastructure development combined with sea level rise and coastal erosion consequently squeezing dune ecosystems into an increasingly narrow coastal strip.[28,49] One option to counter this trend of decreasing coastal space is to move the shoreline seaward through mega-nourishments. Although this is expensive and requires high sediment availability,[50,51] this solution can be preferred in hyperdeveloped coastal zones. Another, more drastic option would be to remove human infrastructure that has been built too close to shore, alleviating pressure on squeezed ecosystems by allowing landward retreat.[52]

Nature management implications

Although our approach and analyses were relatively crude, our survey results from the Netherlands nevertheless suggest that management measures, such as sod cutting, grazing, mowing, and shrub removal, can enhance plant species richness along narrow and wide coasts. A potential reason for this positive effect that we observed along both narrow and wide coasts could be that management not only (partly) counters biodiversity losses as a result of coastal squeeze but also counters the impact of other processes that threaten species richness. Examples of

such processes are the spread of invasive species[53,54] and the "greening" (i.e., increase in vegetation cover) of coastal dunes,[55–57] which homogenize the landscape, disrupt natural morphodynamics,[58] and lead to local extinction of species. In addition to an overall positive effect of management on species richness along the Dutch sandy coast, we found that management measures become increasingly effective as the undisturbed coastal width widens. A potential explanation for this is that large-scale measures, such as remobilization of the coastal dunes,[59] can only be taken in sufficiently wide dune systems, and these large-scale measures may be extra effective in enhancing species richness at the landscape scale. In the southeastern USA, however, we were not able to identify such positive effects of management. Perhaps management is indeed less effective in this region. Alternatively, our inability to detect effects here may also have been caused by (1) the smaller number of transects, (2) the short transect lengths that limit the analysis' sensitivity due to the low number of species richness samples per transect, and/or (3) the fact that fewer intervention types were employed along the US coasts (4 in the USA versus 7 in the NL), while variation in management types along different transects was also limited—potentially due to their short lengths. Overall, in line with previous experimental studies,[41–43] we conclude that targeted management measures can be a vital tool to counteract biodiversity losses in coastal ecosystems. Nevertheless, we also stress that maintaining sufficient coastal width, for instance through the establishment or expansion of nature reserves, should be the first line of defense against coastal ecosystem degradation.

Conclusion

In this study, we show that both plant and habitat diversity nonlinearly depend on coastal width. Therefore, a reduction of coastal width as a result of coastal squeeze by human infrastructure directly reduces plant and habitat diversity. Furthermore, we find a strong positive linear relation between habitat diversity and species richness, which indicates that a diverse habitat assemblage is a prerequisite for species-rich dunes. However, we also find that there is a severe lack of unimpacted coastal space in both the southeastern USA and the Netherlands to accommodate all species-rich habitat assemblages that naturally occur along sandy sea-to-land gradients. This is alarming, as the functioning of ecosystems and their resilience to the increasing threats of climate change and anthropogenic impact generally depend on their biodiversity.[38–40] We therefore conclude that preserving sufficient unimpacted coastal space, for instance through the establishment or expansion of nature reserves, should be a priority for maintaining resilient, biodiverse dune systems. Moreover, as we find that management measures can, at least in part, mitigate biodiversity losses from coastal squeeze, we suggest that strategic interventions can help maintain biodiversity where coastal squeeze cannot be alleviated.

RESOURCE AVAILABILITY

Lead contact

Requests for further information and resources should be directed to, and will be fulfilled by, the lead contact, Eva Lansu (eva.lansu@nioz.nl).

Materials availability

This study did not generate any reagents.

Data and code availability

- Data collected for this study have been deposited at Zenodo and are publicly available at https://doi.org/10.5281/zenodo.15704200.
- All original code has been deposited at Zenodo and is publicly available at https://doi.org/10.5281/zenodo.15704200.
- Any additional information required to reanalyze the data reported in this paper is available from the lead contact upon request.

ACKNOWLEDGMENTS

We would like to thank Adam Hymel, Boris Holtman, Beatriz Marin-Diaz, Joe Morton, and Sydney Willams for their help in the field. We would also like to thank the dune managers for the fieldwork permits and for completing our questionnaires. E.M.L. was funded by the NWO-LLDD grant (17595), S.H. by the OBN grant (OBN-2019-105-DK), V.R. by the NWO-Veni grant (VI.Veni.212.059), M.R. by the ERC-Synergy grant (101071417) and NWO grant (OCENW.M20.169), and T.v.d.H. by the NWO/TTW-Vidi grant (16588).

AUTHOR CONTRIBUTIONS

Conceptualization, E.M.L., T.v.d.H., and V.R.; methodology, E.M.L., C.A., H.S. F., T.v.d.H., and V.R.; data collection, E.M.L., D.W., E.v.W., H.S.F., N.H., and S.T.; formal analysis, E.M.L.; visualization, E.M.L.; supervision, T.v.d.H. and V. R.; writing – original draft, E.M.L.; writing – review & editing, A.M.K., C.A., D.G., E.M.L., H.S.F., L.G., M.R., M.W., S.H., S.d.V., T.v.d.H., and V.R.; funding acquisition, T.v.d.H. and V.R.

DECLARATION OF INTERESTS

The authors declare no competing interests.

STAR★METHODS

Detailed methods are provided in the online version of this paper and include the following:

- KEY RESOURCES TABLE
- EXPERIMENTAL MODEL AND STUDY PARTICIPANT DETAILS
  - Sites description
- METHOD DETAILS
  - Transect selection
  - Sampling procedure
- QUANTIFICATION AND STATISTICAL ANALYSIS
  - Data analysis
  - Species richness mapping
  - Management survey

SUPPLEMENTAL INFORMATION

Supplemental information can be found online at https://doi.org/10.1016/j.cub.2025.09.027.

Received: March 17, 2025
Revised: June 25, 2025
Accepted: September 9, 2025
Published: October 3, 2025

REFERENCES

1. Luijendijk, A., Hagenaars, G., Ranasinghe, R., Baart, F., Donchyts, G., and Aarninkhof, S. (2018). The state of the world's beaches. Sci. Rep. *8*, 6641. https://doi.org/10.1038/s41598-018-24630-6.

2. Martínez, M.L., Psuty, N.P., and Lubke, R.A. (2008). A perspective on coastal dunes. In Ecological Studies 171, 3–10. https://doi.org/10.1007/978-3-540-74002-5_1.
3. Drius, M., Jones, L., Marzialetti, F., de Francesco, M.C., Stanisci, A., and Carranza, M.L. (2019). Not just a sandy beach. The multi-service value of Mediterranean coastal dunes. Sci. Total Environ. *668*, 1139–1155. https://doi.org/10.1016/j.scitotenv.2019.02.364.
4. Barbier, E.B., Hacker, S.D., Kennedy, C., Koch, E.W., Stier, A.C., and Silliman, B.R. (2011). The value of estuarine and coastal ecosystem services. Ecol. Monogr. *81*, 169–193. https://doi.org/10.1890/10-1510.1.
5. Everard, M., Jones, L., and Watts, B. (2010). Have we neglected the societal importance of sand dunes? An ecosystem services perspective. Aquat. Conserv. Mar. Freshw. Ecosyst. *20*, 476–487. https://doi.org/10.1002/aqc.1114.
6. Defeo, O., McLachlan, A., Armitage, D., Elliott, M., and Pittman, J. (2021). Sandy beach social–ecological systems at risk: regime shifts, collapses, and governance challenges. Front. Ecol. Environ. *19*, 564–573. https://doi.org/10.1002/fee.2406.
7. Neumann, B., Vafeidis, A.T., Zimmermann, J., and Nicholls, R.J. (2015). Future coastal population growth and exposure to sea-level rise and coastal flooding - A global assessment. PLoS One *10*, e0118571. https://doi.org/10.1371/journal.pone.0118571.
8. Merkens, J.L., Reimann, L., Hinkel, J., and Vafeidis, A.T. (2016). Gridded population projections for the coastal zone under the shared socioeconomic pathways. Glob. Planet Change *145*, 57–66. https://doi.org/10.1016/j.gloplacha.2016.08.009.
9. van Strien, A.J., Meyling, A.W.G., Herder, J.E., Hollander, H., Kalkman, V.J., Poot, M.J.M., Turnhout, S., van der Hoorn, B., van Strien-van Liempt, W.T.F.H., van Swaay, C.A.M., et al. (2016). Modest recovery of biodiversity in a western European country: The Living Planet Index for the Netherlands. Biol. Conserv. *200*, 44–50. https://doi.org/10.1016/j.biocon.2016.05.031.
10. Sperandii, M.G., Prisco, I., and Acosta, A.T.R. (2018). Hard times for Italian coastal dunes: insights from a diachronic analysis based on random plots. Biodivers. Conserv. *27*, 633–646. https://doi.org/10.1007/s10531-017-1454-1.
11. Sperandii, M.G., Barták, V., Carboni, M., and Acosta, A.T.R. (2021). Getting the measure of the biodiversity crisis in Mediterranean coastal habitats. J. Ecol. *109*, 1224–1235. https://doi.org/10.1111/1365-2745.13547.
12. Lu, Y., Yuan, J., Lu, X., Su, C., Zhang, Y., Wang, C., Cao, X., Li, Q., Su, J., Ittekkot, V., et al. (2018). Major threats of pollution and climate change to global coastal ecosystems and enhanced management for sustainability. Environ. Pollut. *239*, 670–680. https://doi.org/10.1016/j.envpol.2018.04.016.
13. Malavasi, M., Santoro, R., Cutini, M., Acosta, A.T.R., and Carranza, M.L. (2013). What has happened to coastal dunes in the last half century? A multitemporal coastal landscape analysis in Central Italy. Landsc. Urban Plan. *119*, 54–63. https://doi.org/10.1016/j.landurbplan.2013.06.012.
14. Keijsers, J.G.S., Poortinga, A., Riksen, M.J.P.M., and Maroulis, J. (2014). Spatio-temporal variability in accretion and erosion of coastal foredunes in the Netherlands: Regional climate and local topography. PLoS One *9*, e91115. https://doi.org/10.1371/journal.pone.0091115.
15. van Puijenbroek, M.E.B., Limpens, J., de Groot, A.V., Riksen, M.J.P.M., Gleichman, M., Slim, P.A., van Dobben, H.F., and Berendse, F. (2017). Embryo dune development drivers: beach morphology, growing season precipitation, and storms. Earth Surf. Process Landforms *42*, 1733–1744. https://doi.org/10.1002/esp.4144.
16. Galiforni Silva, F., Wijnberg, K.M., de Groot, A.V., and Hulscher, S.J.M.H. (2019). The effects of beach width variability on coastal dune development at decadal scales. Geomorphology *329*, 58–69. https://doi.org/10.1016/j.geomorph.2018.12.012.
17. Hesp, P. (2002). Foredunes and blowouts: initiation, geomorphology and dynamics. Geomorphology *48*, 245–268. https://doi.org/10.1016/S0169-555X(02)00184-8.
18. McLachlan, A. (1991). Ecology of coastal dune fauna. J. Arid Environ. *21*, 229–243. https://doi.org/10.1016/S0140-1963(18)30684-0.
19. Lane, C., Wright, S.J., Roncal, J., and Maschinski, J. (2008). Characterizing environmental gradients and their influence on vegetation zonation in a subtropical coastal sand dune system. J. Coast. Res. *24*, 213–224. https://doi.org/10.2112/07-0853.1.
20. Forey, E., Chapelet, B., Vitasse, Y., Tilquin, M., Touzard, B., and Michalet, R. (2008). The relative importance of disturbance and environmental stress at local and regional scales in French coastal sand dunes. J. Veg. Sci. *19*, 493–502. https://doi.org/10.3170/2008-8-18392.
21. Ruocco, M., Bertoni, D., Sarti, G., and Ciccarelli, D. (2014). Mediterranean coastal dune systems: Which abiotic factors have the most influence on plant communities? Estuar Coast. Shelf Sci. *149*, 213–222. https://doi.org/10.1016/j.ecss.2014.08.019.
22. Haddad, N.M., Brudvig, L.A., Clobert, J., Davies, K.F., Gonzalez, A., Holt, R.D., Lovejoy, T.E., Sexton, J.O., Austin, M.P., Collins, C.D., et al. (2015). Habitat fragmentation and its lasting impact on Earth's ecosystems. Sci. Adv. *1*, e1500052. https://doi.org/10.1126/sciadv.1500052.
23. Pontee, N. (2013). Defining coastal squeeze: A discussion. Ocean Coast. Manag. *84*, 204–207. https://doi.org/10.1016/j.ocecoaman.2013.07.010.
24. Leonard, P.B., Sutherland, R.W., Baldwin, R.F., Fedak, D.A., Carnes, R.G., and Montgomery, A.P. (2017). Landscape connectivity losses due to sea level rise and land use change. Anim. Conserv. *20*, 80–90. https://doi.org/10.1111/acv.12289.
25. Fahrig, L. (2003). Effects of habitat fragmentation on biodiversity. Annu. Rev. Ecol. Evol. Syst. *34*, 487–515. https://doi.org/10.1146/annurev.ecolsys.34.011802.132419.
26. Thompson, S.E.D., Chisholm, R.A., and Rosindell, J. (2019). Characterising extinction debt following habitat fragmentation using neutral theory. Ecol. Lett. *22*, 2087–2096. https://doi.org/10.1111/ele.13398.
27. Pärtel, M., Tamme, R., Carmona, C.P., Riibak, K., Moora, M., Bennett, J.A., Chiarucci, A., Chytrý, M., de Bello, F., Eriksson, O., et al. (2025). Global impoverishment of natural vegetation revealed by dark diversity. Nature *641*, 917–924. https://doi.org/10.1038/s41586-025-08814-5.
28. Lansu, E.M., Reijers, V.C., Höfer, S., Luijendijk, A., Rietkerk, M., Wassen, M.J., Lammerts, E.J., and van der Heide, T. (2024). A global analysis of how human infrastructure squeezes sandy coasts. Nat. Commun. *15*, 432. https://doi.org/10.1038/s41467-023-44659-0.
29. Barbier, E.B., Koch, E.W., Silliman, B.R., Hacker, S.D., Wolanski, E., Primavera, J., Granek, E.F., Polasky, S., Aswani, S., Cramer, L.A., et al. (2008). Coastal ecosystem-based management with nonlinear ecological functions and values. Science *319*, 321–323. https://doi.org/10.1126/science.1150349.
30. Koch, E.W., Barbier, E.B., Silliman, B.R., Reed, D.J., Perillo, G.M.E., Hacker, S.D., Granek, E.F., Primavera, J.H., Muthiga, N., Polasky, S., et al. (2009). Non-linearity in ecosystem services: Temporal and spatial variability in coastal protection. Front. Ecol. Environ. *7*, 29–37. https://doi.org/10.1890/080126.
31. Connor, M.A., Tarvin, S., Quail, M., and Batke, S.P. (2021). Plant diversity and community composition in managed humid coastal dune slacks in NW England. PLoS One *16*, e0256215. https://doi.org/10.1371/journal.pone.0256215.
32. Campos, J.A., Liendo, D., Biurrun, I., Torca, M., and García-Mijangos, I. (2024). Role of plant specialists in fine-scale diversity–area relationships (DARs) in southern European Atlantic coastal dunes. J. Veg. Sci. *35*, 1–12. https://doi.org/10.1111/jvs.70005.
33. Obeso, J.R., and Aedo, C. (1992). Plant-species richness and extinction on isolated dunes along the rocky coast of northwestern Spain. J. Veg. Sci. *3*, 129–132. https://doi.org/10.2307/3236008.
34. Stein, A., Gerstner, K., and Kreft, H. (2014). Environmental heterogeneity as a universal driver of species richness across taxa, biomes and spatial scales. Ecol. Lett. *17*, 866–880. https://doi.org/10.1111/ele.12277.

CellPress
OPEN ACCESS

35. Hortal, J., Triantis, K.A., Meiri, S., Thébault, E., and Sfenthourakis, S. (2009). Island species richness increases with habitat diversity. Am. Nat. *174*, E205–E217. https://doi.org/10.1086/645085.

36. Hutchinson, G.E. (1959). Homage to Santa Rosalia or why are there so many kinds of animals? Am. Nat. *93*, 145–159. https://doi.org/10.1086/282070.

37. Knops, J.M.H., Tilman, D., Haddad, N.M., Naeem, S., Mitchell, C.E., Haarstad, J., Ritchie, M.E., Howe, K.M., Reich, P.B., Siemann, E., et al. (1999). Effects of plant species richness on invasion dynamics, disease outbreaks, insect abundances and diversity. Ecol. Lett. *2*, 286–293. https://doi.org/10.1046/j.1461-0248.1999.00083.x.

38. Oliver, T.H., Heard, M.S., Isaac, N.J.B., Roy, D.B., Procter, D., Eigenbrod, F., Freckleton, R., Hector, A., Orme, C.D.L., Petchey, O.L., et al. (2015). Biodiversity and resilience of ecosystem functions. Trends Ecol. Evol. *30*, 673–684. https://doi.org/10.1016/j.tree.2015.08.009.

39. Peterson, G., Allen, C.R., and Holling, C.S. (1998). Original Articles: Ecological resilience, biodiversity, and scale. Ecosystems *1*, 6–18. https://doi.org/10.1007/s100219900002.

40. Hooper, D.U., Adair, E.C., Cardinale, B.J., Byrnes, J.E.K., Hungate, B.A., Matulich, K.L., Gonzalez, A., Duffy, J.E., Gamfeldt, L., and O'Connor, M. I. (2012). A global synthesis reveals biodiversity loss as a major driver of ecosystem change. Nature *486*, 105–108. https://doi.org/10.1038/nature11118.

41. Deng, C., Daley, T., and Smith, A.D. (2015). Applications of species accumulation curves in large-scale biological data analysis. Quant. Biol. *3*, 135–144. https://doi.org/10.1007/s40484-015-0049-7.

42. Gotelli, N.J., and Colwell, R.K. (2001). Quantifying biodiversity: Procedures and pitfalls in the measurement and comparison of species richness. Ecol. Lett. *4*, 379–391. https://doi.org/10.1046/j.1461-0248.2001.00230.x.

43. Bebber, D.P., Marriott, F.H.C., Gaston, K.J., Harris, S.A., and Scotland, R. W. (2007). Predicting unknown species numbers using discovery curves. Proc. Biol. Sci. *274*, 1651–1658. https://doi.org/10.1098/rspb.2007.0464.

44. Ewald, J. (2003). The sensitivity of Ellenberg indicator values to the completeness of vegetation relevés. Basic Appl. Ecol. *4*, 507–513. https://doi.org/10.1078/1439-1791-00155.

45. Palmer, M.W., and White, P.S. (1994). Scale dependence and the species-area relationship. Am. Nat. *144*, 717–740. https://doi.org/10.1086/285704.

46. Michalet, R., Maalouf, J.P., Choler, P., Clément, B., Rosebery, D., Royer, J.M., Schöb, C., and Lortie, C.J. (2015). Competition, facilitation and environmental severity shape the relationship between local and regional species richness in plant communities. Ecography *38*, 335–345. https://doi.org/10.1111/ecog.01106.

47. Pärtel, M., Szava-Kovats, R., and Zobel, M. (2011). Dark diversity: Shedding light on absent species. Trends Ecol. Evol. *26*, 124–128. https://doi.org/10.1016/j.tree.2010.12.004.

48. Keylock, C.J. (2005). Simpson diversity and the Shannon-Wiener index as special cases of a generalized entropy. Oikos *109*, 203–207. https://doi.org/10.1111/j.0030-1299.2005.13735.x.

49. Vousdoukas, M.I., Ranasinghe, R., Mentaschi, L., Plomaritis, T.A., Athanasiou, P., Luijendijk, A., and Feyen, L. (2020). Sandy coastlines under threat of erosion. Nat. Clim. Chang. *10*, 260–263. https://doi.org/10.1038/s41558-020-0697-0.

50. de Schipper, M.A.D., Ludka, B.C., Raubenheimer, B., Luijendijk, A.P., and Schlacher, T.A. (2021). Beach nourishment has complex implications for the future of sandy shores. Nat. Rev. Earth Environ. *2*, 70–84. https://doi.org/10.1038/s43017-020-00109-9.

51. Powell, E.J., Tyrrell, M.C., Milliken, A., Tirpak, J.M., and Staudinger, M.D. (2019). A review of coastal management approaches to support the integration of ecological and human community planning for climate change. J. Coast. Conserv. *23*, 1–18. https://doi.org/10.1007/s11852-018-0632-y.

52. Neal, W.J., Bush, D.M., and Pilkey, O.H. (2017). Managed retreat. In Encyclopedia of Coastal Science, C. Finkl, and C. Makowski, eds. (Springer), pp. 1–7. https://doi.org/10.1007/978-3-319-48657-4_201-2.

53. Ehrenburg, A., Hagen van der, H., and Terlouw, L. (2008). Amerikaanse vogelkers als invasieve soort in de kustduinen. Levende Nat. *109*, 240–245.

54. Isermann, M. (2008). Expansion of Rosa rugosa and Hippophaë rhamnoides in coastal grey dunes: Effects at different spatial scales. Flora Morphol. Distrib. Funct. Ecol. Plants *203*, 273–280. https://doi.org/10.1016/j.flora.2007.03.009.

55. Jackson, D.W.T., Costas, S., González-Villanueva, R., and Cooper, A. (2019). A global 'greening' of coastal dunes: An integrated consequence of climate change? Glob. Planet Change *182*, 103026. https://doi.org/10.1016/j.gloplacha.2019.103026.

56. Pye, K., and Blott, S.J. (2017). Evolution of a sediment-starved, over-stabilised dunefield: Kenfig Burrows, South Wales, UK. J. Coast. Conserv. *21*, 685–717. https://doi.org/10.1007/s11852-017-0506-8.

57. Pye, K., Blott, S.J., and Howe, M.A. (2014). Coastal dune stabilization in Wales and requirements for rejuvenation. J. Coast. Conserv. *18*, 27–54. https://doi.org/10.1007/s11852-013-0294-8.

58. Gao, J., Kennedy, D.M., and Konlechner, T.M. (2020). Coastal dune mobility over the past century: A global review. Prog. Phys. Geogr. *44*, 814–836. https://doi.org/10.1177/0309133320919612.

59. Arens, S.M., Mulder, J.P.M., Slings, Q.L., Geelen, L.H.W.T., and Damsma, P. (2013). Dynamic dune management, integrating objectives of nature development and coastal safety: Examples from the Netherlands. Geomorphology *199*, 205–213. https://doi.org/10.1016/j.geomorph.2012.10.034.

60. The MathWorks Inc. (2024). MATLAB. https://in.mathworks.com/products/matlab.html.

61. The MathWorks Inc. (2024). Curve Fitting Toolbox. https://nl.mathworks.com/products/curvefitting.html.

62. The MathWorks Inc. (2024). Mapping Toolbox. https://nl.mathworks.com/products/mapping.html.

63. Peel, M.C., Finlayson, B.L., and McMahon, T.A. (2007). Updated world map of the Koppen-Geiger climate classification. Hydrol. Earth Syst. Sci. *11*, 1633–1644. https://doi.org/10.5194/hess-11-1633-2007.

64. Claudino-Sales, V., Wang, P., and Horwitz, M.H. (2008). Factors controlling the survival of coastal dunes during multiple hurricane impacts in 2004 and 2005: Santa Rosa barrier island, Florida. Geomorphology *95*, 295–315. https://doi.org/10.1016/j.geomorph.2007.06.004.

65. Murakami, H., Delworth, T.L., Cooke, W.F., Zhao, M., Xiang, B., and Hsu, P.C. (2020). Detected climatic change in global distribution of tropical cyclones. Proc. Natl. Acad. Sci. USA *117*, 10706–10714. https://doi.org/10.1073/pnas.1922500117.

66. Worldometer. (2023). Worldometers. https://www.worldometers.info/.

67. Verbyla, D.L., and Litvaitis, J.A. (1989). Resampling methods for evaluating classification accuracy of wildlife habitat models. Environ. Manag. *13*, 783–787. https://doi.org/10.1007/BF01868317.

68. Maitner, B.S., Halbritter, A.H., Telford, R.J., Strydom, T., Chacon, J., Lamanna, C., Sloat, L.L., Kerkhoff, A.J., Messier, J., Rasmussen, N., et al. (2023). Bootstrapping outperforms community-weighted approaches for estimating the shapes of phenotypic distributions. Methods Ecol. Evol. *14*, 2592–2610. https://doi.org/10.1111/2041-210X.14160.

STAR★METHODS

KEY RESOURCES TABLE

REAGENT or RESOURCE	SOURCE	IDENTIFIER
Deposited data		
Data and script analyses presented in this study	This paper	Zenodo: https://doi.org/10.5281/zenodo.15704200
Software and algorithms		
MATLAB (version: R2024b)	MathWorks[60]	RRID:SCR_001622; https://nl.mathworks.com/products/matlab.html
MATLAB's Curve Fitting Toolbox	MathWorks[61]	https://nl.mathworks.com/products/curvefitting.html
MATLAB's Mapping Toolbox	MathWorks[62]	https://nl.mathworks.com/products/mapping.html

EXPERIMENTAL MODEL AND STUDY PARTICIPANT DETAILS

Sites description

We conducted the fieldwork in two study areas: the southeastern USA and the Netherlands (Figure 2). A major difference between both study areas is the climate, as the southeastern USA is typified by a humid subtropical climate, whereas the Netherlands has a temperate oceanic climate.[63] While tropical hurricanes play an essential role in re-distributing the sediment of the coastal dunes in the southeastern USA, these do not occur in the Netherlands.[64,65] Both sites are densely populated; 136 people per km^2 in Florida and 508 people per km^2 in the Netherlands.[66]

METHOD DETAILS

Transect selection

To explore the relation between the unimpacted (i.e. free of hard infrastructure such as paved car roads or buildings) coastal width and species richness, we selected coastal dune sites with varying unimpacted coastal widths along the sea-to-land gradient. At each site, we constructed a transect perpendicular to the shoreline, from the beach to the nearest paved road or building with a maximum theoretical length of 25 km.[28] In reality, however, the undisturbed coastal width along the transects was always shorter in our study due to intersecting human infrastructure. A couple of transects that crossed barrier islands did not end due to human infrastructure, but rather because of intersecting the shoreline on the other side. As our transects always ran straight through dune areas, which were managed by nature organizations, they generally crossed typical dune habitats, but occasionally also included less typical habitats, such as salt marsh vegetation in nearshore dune valleys and needleleaf forests in back dunes. For consistency, we did not exclude any habitat type and sampled the full undisturbed length of each transect (see sampling procedure). In the southeastern USA, sites were generally more truncated by infrastructure than in the Netherlands. Therefore, transects ranged from 100 to 1500 m in the USA, whereas the Dutch transects ranged from 200 to 4300 m (Table S1). 12 transects were located along the South Atlantic Bight in the USA and 35 along the North Sea coast of the Netherlands (Figure 2).

Sampling procedure

At each transect, we followed the natural chronosequence of habitats by sampling along the sea-to-land gradient. To capture all habitats along the transects (i.e. not skip a habitat), we started by sampling the embryo dune zone, followed by the foredunes. Next, behind the foredunes, sampling stations were spaced 100 meters apart. At each sampling station, we sampled the vegetation composition. Specifically, we identified the plant species and visually estimated their cover in percentages. At each station, herb and moss species were identified in a 2*2m plot, shrubs in a 5*5m plot, and trees in a 10*10m plot. In total, we performed vegetation recordings at 108 stations along 12 transects in the USA and at 506 stations along 35 transects in the Netherlands. We conducted the fieldwork between 12-09-2022 and 22-09-2022 in the USA and between 18-05-2021 and 03-09-2021 in the Netherlands.

QUANTIFICATION AND STATISTICAL ANALYSIS

Data analysis

Based on the vegetation recordings, we assigned a habitat type to each sampling station. We applied criteria based on the presence of distinctive/characteristic species, and the moss, herb, shrub and tree cover (Table S2). Next, to investigate whether species

richness accumulates in a nonlinear fashion with increasing distance from the coastline as hypothesized, we fitted an asymptotic *Von Bertalanffy growth* function defined as (Bertalanffy, 1957):

$$Y(X) = p\left(1 - e^{-kX}\right) \quad \text{(Equation 1)}$$

where Y is the number of species at distance to sea X (m), p is the asymptotic value of the number of species, and k is the relative rate of species accumulation over distance to sea (proportion of the asymptotic value per m). We fitted the function to describe the average species accumulation per site (Figures 3A and 3B), the average habitat accumulation per site (Figure S2), and the species accumulation per transect (Figure S1). Next, to test whether the relations were indeed nonlinear, we compared the quality of the fit with a linear regression model without intercept using the Akaike's Information Criterion. To identify the extent to which sampling effort affected our results,[43] we performed a sensitivity analysis. Specifically, we doubled the intervals between the sampling stations and refitted the species and habitat accumulation curves. Differences between the original and these newly obtained curves indicate the sensitivity of our outcomes to sampling effort. All regression analyses were performed using MATLAB's Curve Fitting Toolbox.[61]

Species richness mapping

We estimated the current species richness status for both study areas based on the calculated coastal width-species richness relationships. To achieve this, we used the infrastructure-free coastal space – the distance between the shoreline (OpenStreetMap, 2023) and the nearest human infrastructure – as a measure for the unimpacted coastal width of the dune area (see Lansu et al.[28] for further methodological details). First, along every kilometer of sandy shore in the Netherlands and in the southeastern USA, we computed the cross-shore distance between the sandy shoreline and the nearest building or paved car road. Secondly, we calculated what species richness can potentially be reached, within the local infrastructure-free coastal width. For our calculations, we used the averaged distance to sea – species richness relationships (Equation 1) per region. Finally, we analyzed what parts of the sandy coastline have a protected status (World Database on Protected Areas). For simplicity and as a conservative approach, we considered a sandy coast protected when a protected area (local, state, federal, or NGO protection) encompassed the first 200 m of habitat from the sea. The maps were created with MATLAB's Mapping Toolbox.[62]

Management survey

To assess the relative influence of dune management and transect length on species richness, we conducted a survey among nature managers per transect (Table S3) and included the outcomes together with transect length in multiple linear regression analyses. We defined management intensity as the number of different interventions performed within the vicinity (250 m) of the transect over the last 20 years. In the Netherlands, we included 5 interventions commonly applied to reset vegetation succession: sod cutting, mowing (vegetation is cut and removed), grazing, flailing (vegetation is not cut, but beaten off and left behind), and removal of shrubs. We also included 2 interventions commonly implemented to increase sediment dynamics (i.e. remobilization): creation of notches in the foredunes and the creation of blowouts. The score of management intensity ranged between 0 (no interventions) and 7 (all specified interventions performed). In the USA, we included 3 interventions commonly applied to reset vegetation succession: invasive plant species removal (including shrubs), invasive animal species removal, and grazing. We also included one applied intervention to increase sediment dynamics: controlled burning. The score of management intensity ranged between 0 (no interventions) and 4 (all specified interventions performed).

Prior to performing the multiple regression analyses, we standardized sampling effort for species richness across transects, using a bootstrapping approach.[67,68] Specifically, we randomly subsampled 2 plots per transect in the USA and 3 plots per transect in the Netherlands, which was the minimum number of plots obtained for a transect in each region. We repeated this subsampling approach 10,000 times per transect and calculated the average *total* number of species present in each subsample. Next, we fitted a stepwise linear regression model to the obtained species richness data, removing terms based on the Akaike's information criterion. We included the following independent variables to explain the average number of species per transect (calculated with our bootstrapping approach): transect length (km), number of management interventions per kilometer, and the interaction between transect length and number of management interventions per kilometer.

Current Biology, Volume *35*

Supplemental Information

How human infrastructure threatens biodiversity by squeezing sandy coasts

Eva M. Lansu, Hallie S. Fischman, Christine Angelini, Nadia Hijner, Luc Geelen, Dick Groenendijk, Solveig Höfer, Annemieke M. Kooijman, Max Rietkerk, Sten Tonkens, Sierd de Vries, Martin Wassen, Evaline van Weerlee, Daniël Wille, Valérie Reijers, and Tjisse van der Heide

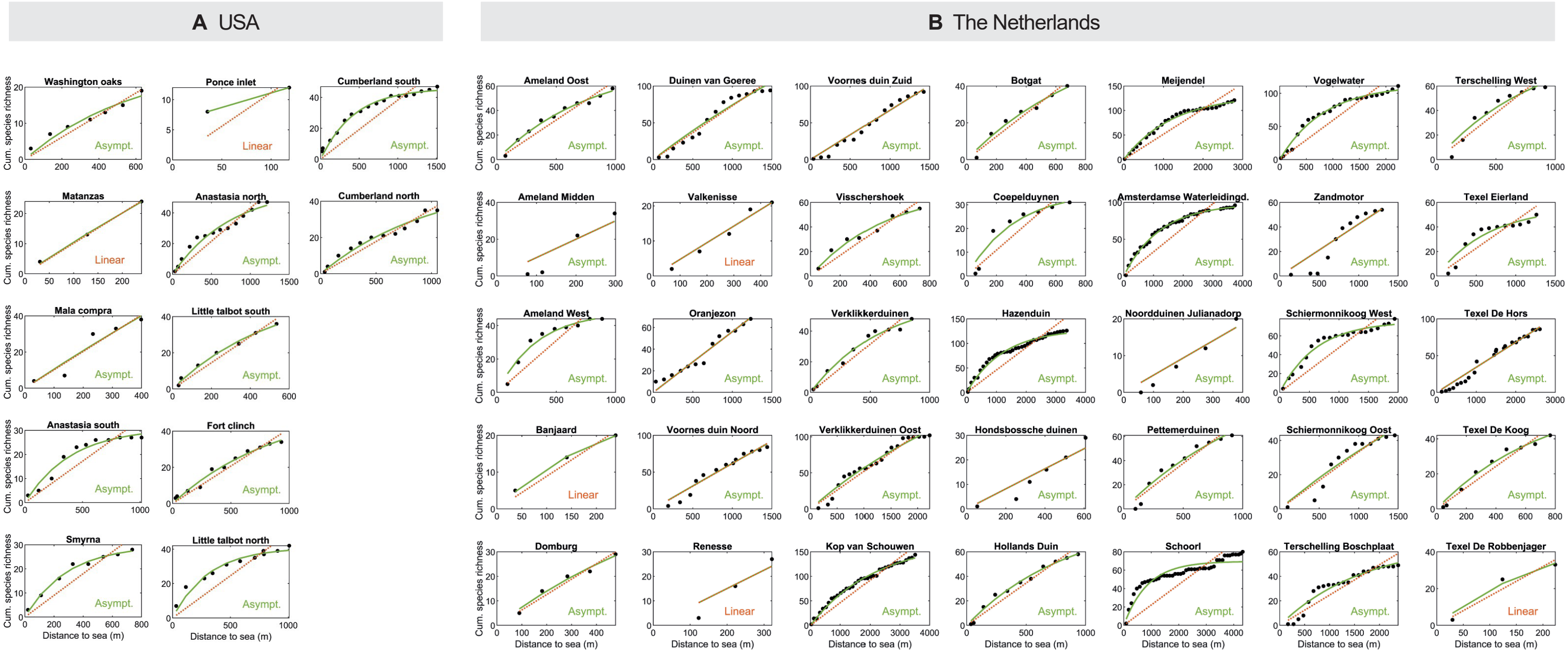

Figure S1. Species accumulation curves per transect. Related to Figure 3. Species accumulation curves of each of the studied transects in southeastern USA (**A**) and the Netherlands (**B**), with the linear regressions (without intercept) displayed orange and the asymptotic regressions displayed green. We selected the best model for each transect based on AIC.

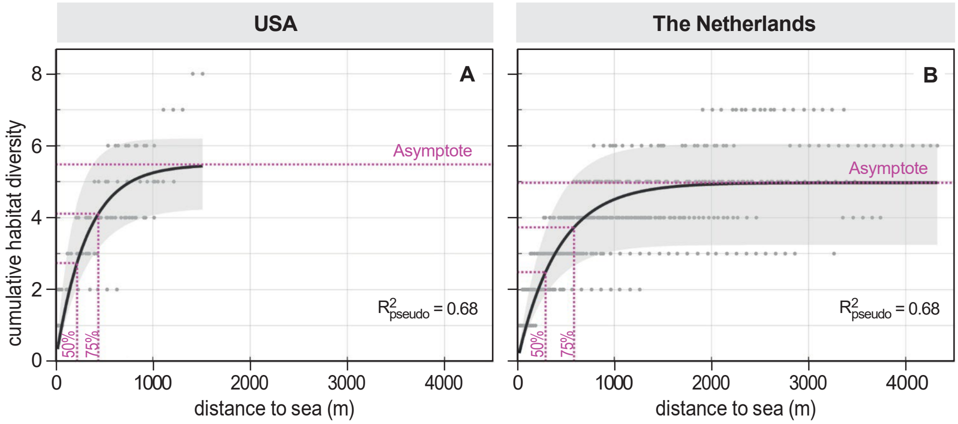

Figure S2. Habitat accumulation curves. Related to Figure 3. Habitat accumulation along the transects in southeastern USA (**A**) and the Netherlands (**B**).

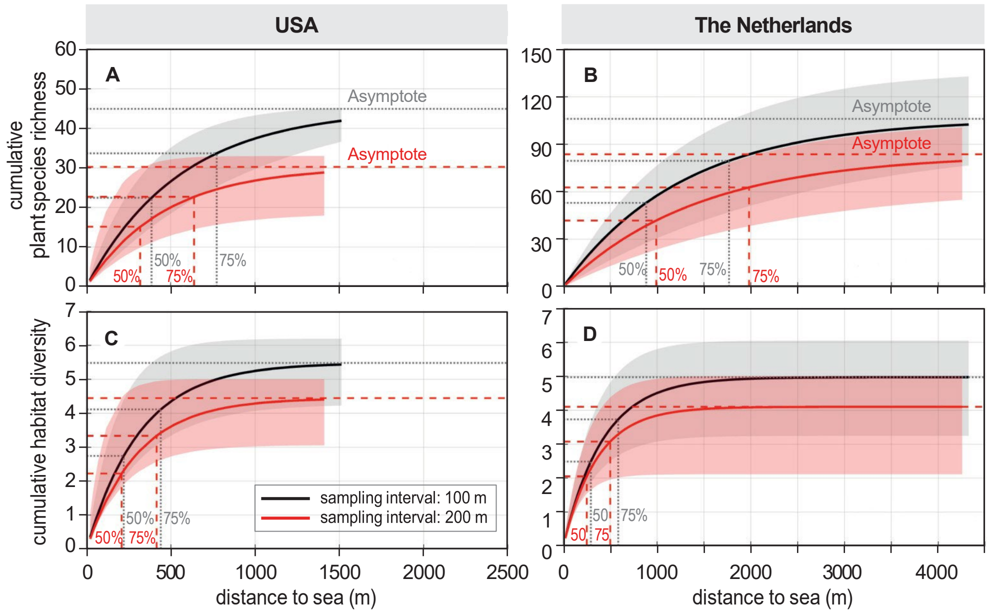

Figure S3. Sensitivity analysis to explore the effect of sampling effort. Related to Figure 3. By deleting every other sample station location along the transects, we increased the sampling interval from 100 m (black line) to 200 m (red line). Subsequently, the species accumulation curves were computed again for the southeastern USA (**A**) and the Netherlands (**B**). In addition, the habitat accumulation curves were computed for both the 100 m and the 200 m interval for the southeastern USA (**C**) and the Netherlands (**D**).

Transects	Transect length (m)	Latitude	Longitude	Species richness	Habitat diversity
Cumberland North	1052	30.78705	–81.45524	49	5
Cumberland South	1512	30.71845	–81.44907	69	8
Fort Clinch	933	30.69228	–81.42811	44	6
Little Talbot North	1008	30.46248	–81.41209	61	5
Little Talbot South	535	30.43905	–81.40802	46	4
Anastasia North	1216	29.90410	–81.27808	67	5
Anastasia South	1013	29.86415	–81.26710	34	4
Matanzas	240	29.70896	–81.22754	38	3
Washington Oaks	631	29.63439	–81.19835	25	2
Mala Compra	399	29.61447	–81.18939	43	3
Ponce Inlet	118	29.08091	–80.92157	19	2
Smyrna	736	29.07317	–80.91648	35	5
Schiermonnikoog Oost	1457	53.509815	6.314283	43	4
Schiermonnikoog West	1940	53.499904	6.167723	78	5
Ameland Oost	974	53.467320	5.910009	58	5
Ameland Midden	298	53.462008	5.801228	34	3
Ameland West	886	53.456472	5.620974	44	3
Terschelling Boschplaat	2374	53.429402	5.411078	49	5
Terschelling West	915	53.401986	5.244782	59	5
Texel De Robbenjager	223	53.182479	4.859673	33	2
Texel Eierland	1262	53.174530	4.832611	50	2
Texel De Koog	766	53.087154	4.740117	42	3
Texel De Hors	2610	52.993224	4.729137	86	7
Noordduinen Julianadorp	378	52.882804	4.708658	20	4
Botgat	677	52.865850	4.702635	40	4
Pettemerduinen	910	52.784281	4.665458	61	5
Hondsbossche duinen	608	52.718910	4.636628	29	3
Schoorl	4325	52.700660	4.635027	80	6
Vogelwater	2231	52.586610	4.612882	111	6
Hazenduin	3364	52.534578	4.599579	126	7
Amsterdamse Waterleidingduinen	3741	52.315653	4.484371	96	4
Hollands Duin	937	52.272737	4.451405	58	5
Coepelduynen	693	52.225119	4.410146	31	4
Meijendel	2790	52.155730	4.338499	121	5
Zandmotor	1294	52.049719	4.181619	54	4
Voornes duin Noord	1440	51.910601	4.048451	84	5
Voornes duin Zuid	1425	51.891549	4.030823	92	5
Duinen van Goeree	1479	51.836959	3.942686	94	6
Visschershoek	733	51.788766	3.863601	55	4
Renesse	321	51.743192	3.766741	27	2
Verklikkerduinen Oost	2224	51.733573	3.712875	102	4
Verklikkerduinen	849	51.720931	3.690537	48	4
Kop van Schouwen	3512	51.691457	3.681640	144	6
Banjaard	239	51.594930	3.665045	20	2
Oranjezon	1232	51.592296	3.567357	68	5
Domburg	477	51.574618	3.517485	29	3
Valkenisse	441	51.485731	3.504876	21	3

Table S1. Details of transects. Related to Figure 2. Details of transects in southeastern USA and the Netherlands.

**Southeastern USA**	
Broadleaf forest	– Tree cover > 25% AND:<br>○ EITHER: The dominant tree species is *Quercus virginiana*<br>○ OR: Needleleaf tree cover < 50% AND Palm tree cover < 50%
Salt Marsh	– Salt loving species cover > 25% OR (salt loving species cover / total herb cover) > 50%
Needleleaf forest	– Tree cover > 25%<br>– AND Needleleaf tree cover > 50%
Open dune grassland	– Tree cover < 25% AND Shrub cover < 25% AND (Shrub + Tree cover) < 25%<br>– AND total vegetation cover < 50%
Palm forest	– Tree cover > 25%<br>– AND Palm tree cover > 50%
Shrubs	– Shrub cover > 25% OR (Shrub + Tree cover) > 25%
Closed dune grassland	– Tree cover < 25% AND Shrub cover < 25% AND (Shrub + Tree cover) < 25%<br>– AND: total vegetation cover > 50%
Foredune	– The seaward–most established (non–incipient) dune; based on field observation
Embryo dune	– Incipient dune on the beach; based on field observation
**The Netherlands**	
Heather	– Tree cover < 25*% AND:*<br>○ EITHER: *Empetrum nigrum* is present<br>○ OR: *Calluna vulgaris* > 25% OR (*Calluna vulgaris* / total herb cover) > 50%
Salt Marsh	– Based on field observation of salt marsh
Needleleaf forest	– Tree cover > 25%<br>– AND: Needleleaf tree cover > 50%
Broadleaf forest	– Tree cover > 25%<br>– AND: Needleleaf tree cover < 50%
Dune Slack	– Dune slack species cover > 25% OR (dune slack species / total herb cover) > 50%<br>– OR: Based on field observation
Closed dune grassland	– Tree cover < 25% AND Shrub cover < 25% AND (Shrub + Tree cover) < 25%<br>– AND: total vegetation cover > 50%
Shrubs	– Shrub cover > 25% OR (Shrub + Tree cover) > 25%
Open dune grassland	– Tree cover < 25% AND Shrub cover < 25% AND (Shrub + Tree cover) < 25%<br>– AND: total vegetation cover < 50%
Foredune	– The seaward–most established (non–incipient) dune; based on field observation
Embryo dune	– Incipient dune on the beach; based on field observation

Table S2. Habitat criteria. Related to STAR Methods. The criteria used to assign habitat types to the sample stations in southeastern USA and the Netherlands.

Transects in southeastern USA	Number of managements interventions	Controlled burns	Invasive plant species removal	Invasive animal species removal	Grazing
Cumberland North	2	0	0	1	1
Cumberland South	3	0	1	1	1
Fort Clinch	2	0	1	1	0
Little Talbot North	2	0	1	1	0
Little Talbot South	2	0	1	1	0
Anastasia North	3	1	1	1	0
Anastasia South	3	1	1	1	0
Matanzas	2	0	1	1	0
Washington Oaks	1	0	1	0	0
Mala Compra	1	0	1	0	0
Ponce Inlet	1	0	1	0	0
Smyrna	1	0	1	0	0

Transects in The Netherlands	Number of management interventions	Sod cutting	Mowing	Flailing	Grazing	Shrub removal	Creation of notches	Creation of blow- outs
Schiermonnikoog Oost	0	0	0	0	0	0	0	0
Schiermonnikoog West	3	0	1	1	0	1	0	0
Ameland Oost	3	1	0	0	0	1	1	0
Ameland Midden	1	0	0	0	0	0	1	0
Ameland West	0	0	0	0	0	0	0	0
Terschelling Boschplaat	0	0	0	0	0	0	0	0
Terschelling West	3	1	1	0	1	0	0	0
Texel De Robbenjager	1	0	1	0	0	0	0	0
Texel Eierland	4	0	1	1	1	0	0	1
Texel De Koog	2	0	0	0	1	1	0	0
Texel De Hors	1	0	1	0	0	0	0	0
Noordduinen Julianadorp	3	0	1	0	0	1	0	1
Botgat	0	0	0	0	0	0	0	0
Pettemerduinen	4	0	1	1	1	1	0	0
Hondsbossche duinen	0	0	0	0	0	0	0	0
Schoorl	5	1	0	1	1	1	0	1
Vogelwater	5	0	1	0	1	1	1	1
Hazenduin	4	0	1	0	1	1	0	1
Amsterdamse Waterleidingd.	6	1	1	0	1	1	1	1
Hollands Duin	2	0	0	0	0	1	0	1
Coepelduynen	0	0	0	0	0	0	0	0
Meijendel	5	1	1	0	1	1	1	0
Zandmotor	3	0	0	0	1	1	1	0
Voornes duin Noord	1	0	0	0	0	1	0	0
Voornes duin Zuid	3	0	1	0	1	1	0	0
Duinen van Goeree	4	0	1	1	1	1	0	0
Visschershoek	5	0	1	0	1	1	1	1
Renesse	2	0	0	0	1	1	0	0
Verklikkerduinen Oost	3	0	1	0	1	1	0	0
Verklikkerduinen	3	1	0	0	1	1	0	0
Kop van Schouwen	4	1	0	0	1	1	1	0
Banjaard	0	0	0	0	0	0	0	0
Oranjezon	3	1	0	0	1	1	0	0
Domburg	3	0	1	1	0	1	0	0
Valkenisse	1	0	1	0	0	0	0	0

Table S3. Result of management questionnaires. Related to Table 1. Result of questionnaires about management interventions targeted at resetting vegetation succession and increasing sediment dynamics in southeastern USA and in the Netherlands.

The Netherlands

Linear regression model: species richness ~ 1 + transect length * number of interventions per km transect

	St. Estimate	SE	P value
**Intercept**	0.11	0.15	0.44
**Transect length**	0.62	0.15	2.1E-04
**Number of interventions per km transect**	0.60	0.19	3.8E-03
**Transect length: Number of interventions per km transect**	0.48	0.21	3.1E-02

Number of observations: 35

R-squared: 0.40

Adjusted R-Squared: 0.34

Table S4. Regression model. Related to Table 1. Linear regression model to explain species richness of the Dutch transects. The species richness was subsampled to ensure uniform sampling effort across transects. To approach a normal distribution of regression residuals, the explaining variables were square root transformed. Additionally, all variables were standardized using z-score transformation.
